# Novel Biomarkers Predictive of Diabetic Charcot Foot—An Overview of the Literature

**DOI:** 10.3390/life12111944

**Published:** 2022-11-21

**Authors:** Anca Bobircă, Anca Emanuela Musetescu, Anca Bordianu, Anca Pantea Stoian, Teodor Salmen, Dan-Cristian Marinescu, Cristina Alexandru, Alesandra Florescu, Raluca Radu, Sebastian Isac, Traian Patrascu, Dragos Serban, Florin Bobircă

**Affiliations:** 1 Internal Medicine and Rheumatology Department, Dr. Ion Cantacuzino Clinical Hospital, 011437 Bucharest, Romania; 2 Internal Medicine and Rheumatology Department, Carol Davila University of Medicine and Pharmacy, 050474 Bucharest, Romania; 3 Rheumatology Department, University of Medicine and Pharmacy Craiova, 200349 Craiova, Romania; 4 Plastic Surgery Department, Bagdasar-Arseni Emergency Hospital, 041915 Bucharest, Romania; 5 Department of Diabetes, Nutrition and Metabolic Diseases, Carol Davila University of Medicine and Pharmacy, 050474 Bucharest, Romania; 6 Doctoral School, Carol Davila University of Medicine and Pharmacy, 050474 Bucharest, Romania; 7 Department of Immunogenetics and Virology, Fundeni Clinical Institute, 022328 Bucharest, Romania; 8 Clinics of Diabetes, Nutrition and Metabolic Disease, 610136 Piatra Neamt, Romania; 9 Department of Physiology and Neuroscience, Carol Davila University of Medicine and Pharmacy, 050474 Bucharest, Romania; 10 Surgery Department, Dr. Ion Cantacuzino Clinical Hospital, 011437 Bucharest, Romania; 11 Surgery Department, Carol Davila University of Medicine and Pharmacy, 050098 Bucharest, Romania; 12 Forth General Surgery Department, Carol Davila University of Medicine and Pharmacy, 050474 Bucharest, Romania

**Keywords:** Charcot diabetic foot, biomarkers, neuropathy, inflammation

## Abstract

*Background*: Although Charcot diabetic foot (CDF) is a frequent complication of diabetic neuropathy, less is known about the possibility of its early prevention. *Methods*: A review of the original articles published in English, using the “biomarkers AND Charcot’s foot” criterion, resulted in 33 articles from the PubMed database and seven articles from the Web of Science database. The five duplicates were eliminated, and two independent reviewers selected the most relevant articles, leaving a total of 21 articles. *Results*: The biomarkers identified are exhaustively described, related to the system of advanced glycation end products (AGEs) and their soluble receptors (sRAGE), inflammatory cascade, osteoclastogenesis, and, respectively, osteoblastic activity. *Conclusions*: This article highlights the importance of potential early identifiable biomarkers that can lead to microstructural changes in the affected bones.

## 1. Introduction

Diabetes mellitus (DM) is considered one of the most common disorders in modern society, whose prevalence has risen globally, along with the associated complications brought on by the physiopathological progression of the disease. In 2015, diabetes prevalence was estimated to be around 415 million people worldwide. However, by 2040, that number is projected to rise to nearly 642 million, a 55% increase, leading the scientific community to classify diabetes as a global epidemic [1]. Being clinically regarded as a chronic metabolic disease, diabetes is characterized by elevated blood glucose levels and vascular complications affecting large and small vessels, pathological conditions known as macro- and microangiopathies, respectively [2]. One of the most severe complications is diabetic foot syndrome, known as Charcot diabetic foot (CDF) or Charcot neuropathic arthropathy (CNA), because there is a higher risk of disability, and it can happen in a short period of time [3,4,5].

Charcot neuropathic arthropathy represents a severe complication that implies a non-reducible foot deformity placing patients with distal peripheral neuropathy at significantly higher risks for developing chronic neuropathic foot ulcers, ultimately leading to major lower extremity amputation and even death [3,6]. William Musgrave first described CNA in 1703, but the condition was further elaborated in 1868 by Jean-Martin Charcot, a French neurologist and professor of anatomical pathology at Pitié-Salpêtrière Hospital in Paris, who studied patients with tabes dorsalis (myelopathy due to syphilis). The neuropathically mediated destruction of the bones and joints of the foot can lead to a rocker-bottom collapse deformity, primarily clustered around the tarsometatarsal and navicular-cuneiform joints [4,5].

### 1.1. Epidemiology

Amongst the global diabetic population, Charcot neuropathic arthropathy is an uncommon finding. Nonetheless, because the diagnostic criteria for neuroarthropathy might vary from one series to another and reported series frequently come from specialized facilities with exposure to the most severe diabetic patient cases, it is difficult to pinpoint the incidence of this condition [7,8,9]. For example, a prospective study in a British population of 205,033 patients with diabetes identified 90 cases of active Charcot arthropathy, translating into a prevalence of 0.04 percent [10]. Additionally, a study of 561,597 patients with diabetes, primarily diagnosed with type 2, from the US Department of Veterans Affairs that focused on inpatient and outpatient datasets for the year 2003 revealed a 0.12% frequency of newly diagnosed diabetic (Charcot) neuroarthropathy, acknowledging obesity and other contributing factors as capable of increasing the health-associated risks [9]. Other research reveals that the prevalence of Charcot neuroarthropathy in a general diabetic population varies between 0.1 and 7.5%. In the case of diabetic patients suffering from apparent peripheral neuropathy, the prevalence of Charcot’s foot increases up to 35% [5]. Notably, the risk associated with Charcot neuroarthropathy does not seem to be related to the clinical type of diabetes mellitus, whether type I or II. Moreover, there have been cases reporting that the bilateral involvement of the feet varies between 9 and 75% [5].

### 1.2. Pathogenesis

Although there has been some significant new research on the pathophysiology of CDF in the last decade, the exact mechanism of this condition remains unknown. The disease development is believed to be multifactorial, with the main contributors being mechanical stress and injuries, vascular involvement, diabetic peripheral and autonomic neuropathy, and metabolic bone abnormalities caused by an inflammatory reaction [11]. The reduction in proprioception brought on by the progression of peripheral neuropathy may result in higher levels of ligamentous laxity, increased joint instability, and a greater range of motion, making the patient more vulnerable to small mechanical stress [11]. The consequent transformations in the physical biomechanics of the foot determine secondary weight-bearing alterations, causing abnormal plantar pressure and further adding to the subsequent localized injuries. Additionally, autonomic neuropathy physiopathology determines vasomotor consequences, including the formation of various arteriovenous shunts arising in a hyperemic state of the lower limb. Finally, bone resorption occurs due to increased blood flow, leading to further deformities. Thus, the bone is more susceptible to fracture [11].

### 1.3. Inflammatory Signaling

It is a common belief that inflammation is a critical element of the pathogenesis of Charcot neuroarthropathy. The so-called “inflammatory theory” emphasizes the pathogenic significance of local inflammatory processes, consequently increasing the expression of pro-inflammatory cytokines, most important in this process being IL-1β, IL-6, and TNF-a [12]. Although this disease’s detailed physiopathology remains unclear, several mechanisms are presumed to have a defining impact on its evolution. The underlying primary condition remains polyneuropathy, which, in the background, enables the cytokine-associated effects to promote an enhanced osteoclastic activity, determining an osteoclast–osteoblast imbalance, as demonstrated by surgical samples taken from a series of patients with neuropathic arthropathy [13]. In the case of CNA patients, as the inflammatory circumstances progress, osseous tissue turnover imbalances begin to emerge [14]. Studies that followed the venous-arterial flux of IL-6 saw a significant upsurge in the affected foot of patients with CNA versus their healthy foot, indicating that IL-6 might be produced at the site of the affliction [15]. At the same time, elevated serum levels of TNF-α, IL-6, or C-reactive protein (C-RP) have also been detected in patients diagnosed with acute CAN preliminary to administrating treatment. There are noticeable decreases in these pro-inflammatory cytokines following casting therapy, the recommended course of treatment for this illness [16]. Moreover, serum TNF-α and IL-6 have been documented to correlate with bone turnover markers such as C-terminal telopeptide positively and serum osteoprotegerin (OPG) levels at initial patient presentation but not during therapeutic measures [16].

The outsized inflammatory reaction in response to sometimes minor traumatic events may trigger an inflammatory overflow via an augmented expression of pro-inflammatory cytokines, especially interleukin (IL)-1, resulting in a marked osteoclastic activity. This series of events leads to osteolysis, and fractures, which can further potentiate the inflammatory cycle through the means of the receptor activator of the nuclear factor kappa-b ligand (RANKL) pathway. Laboratory studies have shown that both receptor activators of nuclear factor (NF) kappa B (RANK) ligand (RANKL)-dependent and -independent pathways may have a role in overpowering the bone resorption mechanisms [17].

### 1.4. Cytokine Balance

The progression of Charcot neuroarthropathy can be significantly impacted by ineffective control of the balance between cytokines with pro- and anti-inflammatory activities. Under physiological conditions, there is a continuous remodeling process, the system homeostasis being ensured by the optimal interaction between osteoclasts that control bone resorption and the osteoblasts responsible for the osseous tissue formation [18]. The RANK/RANKL pathway is the primary mechanism contributing to this equilibrium. The link between RANK/RANKL and osteoprotegerin (OPG), a crucial component of bone resorption, serves as the foundation for the process. The RANKL belongs to the TNF superfamily and can be found in the cellular membrane of osteoblasts as well as bone marrow stromal cells. Its receptor, RANK, is expressed in the preosteoclast membrane and belongs to the TNF receptor superfamily [19].

**Figure 1:**
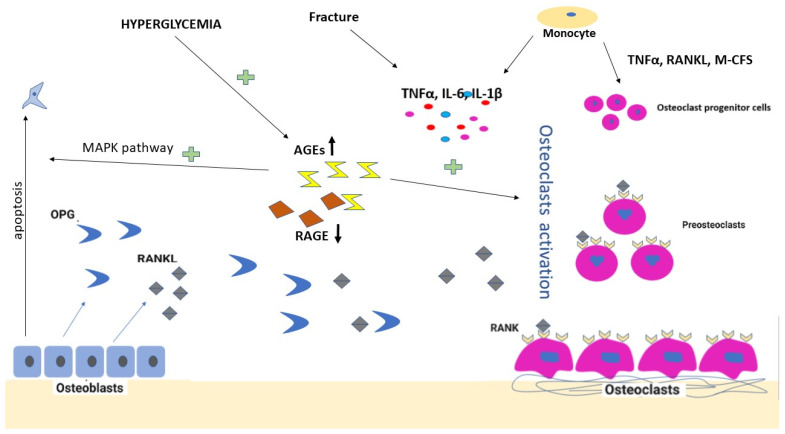
Diagram representation of the mechanism of osteoclast activation. RANK—receptor activator of nuclear factor κB; RANKL—receptor activator of nuclear factor κB ligand; OPG—osteoprotegerin; M-CFS—monocyte colony-stimulating factor; IL—interleukin; TNF-α—tumor necrosis factor-alpha; AGEs—advanced glycation end products; RAGE—receptor for advanced glycation end products.

RANKL holds a primary role in the differentiation and activation of osteoclast precursors as they develop into mature osteoclasts (Figure 1). This molecule can exist both as a membrane-bound protein formed by osteoblasts and activated T cells, as well as a soluble protein that can be detected in blood serum [18]. RANKL’s biological functions are closely related to its interaction with the RANK receptor, located on the surface of preosteoclasts or mature osteoclasts. The operating mechanism involves the binding of RANKL to the RANK receptor, which further initiates a cascade cell signaling that promotes the recruitment of TRAF factors (TRAF2, TRAF5, and TRAF6) to the cytoplasmic domain of RANK, thus further triggering the activation of nuclear transcription factors NK-kB and JNK, which, in turn, stimulates preosteoclasts to differentiate into osteoclasts [14].

Osteoclast activation is due to increased RANKL in the RANK/RANKL/OPG axis. Hyperglycemia induces increased levels of AGEs and reduced levels of RAGE, which stimulates the bone resorption process by osteoblast apoptosis and osteoclast activation. Microtrauma and fracture perpetuate the inflammation with increased levels of cytokines (TNF-α, Il-6, IL1β, etc.), further influencing bone resorption. On the one hand, monocytes are involved in the inflammatory mechanism by expressing cytokines, and on the other hand, they can differentiate into osteoclast progenitor cells under the effect of TNF-α, RANKL, and M-CFS.

However, the ability of OPG to act as a soluble RANKL decoy receptor and prevent RANKL from interacting with RANK can limit osteoclast differentiation [14]. In CDF, the RANK/RANKL/OPG equilibrium is disturbed, with an increase in RANKL, leading to increased osteoclastogenesis (Figure 1) [3]. Otherwise, if the balance is tilted toward an excess of OPG in case of a reduction in RANKL, the resulting process would potentiate osteogenesis, making the RANK/OPG axis invaluable for healthy bone tissue regulation mechanisms [3].

Further cytokinetic agents have been studied to understand the underlying molecular interactions in bone regulation more deeply. One such pathway is Wnt, which is believed to be an essential regulator during the embryonic development of bone tissue, mechanical loading or unloading of the skeleton, bone growth, bone remodeling, and fracture repair. This signaling stimulates osteoblast activation and proliferation from their respective progenitor cells [20]. Moreover, there is a crosslink between the Wnt pathway, which serves an anabolic role for the bone tissue, and the RANKL/OPG pathway, which has contrasting catabolic properties [20]. The Wnt pathway regulates osteoclast differentiation and activation by directly influencing osteoblasts, the osteoclast recruitment process, and bone remodeling initiation [20]. Consistent off-loading therapy is thought to normalize the suppression of the Wnt-signaling pathway [3]. Moreover, this effect is thought to be caused by the proliferation of a receptor for advanced glycation end products (RAGE), which also plays a significant role in hyperglycemia. [3]. Additionally, advanced glycation end products (AGEs) are determining factors regarding collagen crosslinking and arterial wall stiffening, and, in the case of diabetic patients diagnosed with Charcot arthropathy, the oxidative stress caused by these molecules can perpetuate the inflammatory cycle. In patients with persistent hyperglycemia, RAGE levels are reduced, and in turn, AGE levels are increased, therefore controlling the amount of total RANKL available for inflammation signaling [3].

The Wnt/β-catenin signaling pathway also influences bone anabolic activity. In DM patients, its regular functions seem to be disrupted, involving the sclerostin, dickkopf-1 (Dkk-1), Wnt ligand-1 (Wnt-1), and Wnt inhibitory factor-1 (Wif-1). In particular, sclerostin and Dkk-1 serum levels are elevated in diabetic patients, linking the Wnt pathway to higher fracture risk [21]. On the other hand, sclerostin and Dkk-1 levels in Charcot patients are lower than those found in DM patients, resulting in an augmented bone anabolic activity, probably due to bone destruction [20]. Furthermore, some experimental studies have demonstrated that sclerostin and Dkk-1 inhibition enhance bone fracture healing [22,23].

In patients with CDF, inflammation is highly perpetuating at the synovium level since it is richly innervated, and neuropathy is the primary mechanism of Charcot neuroarthropathy. In the rheumatoid synovium during pannus formation, Cadherin-11, a molecular signaling glycoprotein that can be found on cell membranes, is thought to have an essential role [24]. Further research revealed that the synovium could demonstrate invasive behavior if TNF-α is added to the culture, as was the case with probes obtained from rheumatoid arthritis patients [24].

### 1.5. Additional Contributing Factors

The CDF’s physical degradation and mechanical instability may also be influenced by several additional factors related to the bone architecture itself. In the particular case of CNA, the articular surfaces are structurally impaired, pertaining to the scarcity of cartilaginous and fibro-osseous tissue [25]. Additionally, a significant reduction in the sympathetic nerve fiber count in osseous tissue is demonstrated by the depletion of vasoactive intestinal peptide (VIP) and decreased osseous resorption [26]. Moreover, patients with CNA had similar levels of the P substance compared to those with osteoarthritis [26]. Thus, particular neuropeptide dysregulation, such as decreased VIP values in combination with relatively normal levels of P substance, is thought to support the mechanism of persistent inflammation in CDF [27].

Other agents, such as the calcitonin gene-related peptide (CGRP) and nitric oxide synthase (eNOS), can exert effects during the pathogenesis of CNA. Patients diagnosed with Charcot neuroarthropathy display lowered activity of the enzyme nitric oxide synthase (eNOS), subsequently diminishing nitric oxide (NO) concentrations. Although NO is a ubiquitous molecule, at the articular level, experiments in murine models revealed that it could yield the apoptosis of osteoclastic precursor cells [28]. Regarding the human osseous system, NO mediates the flow rate through the periosteocytic canaliculi and impacts the cellular apoptosis mechanisms of the bone tissue. Thus, low NO levels cannot effectively mediate the destructive inflammatory processes, so it promotes bone resorption [29]. As for CGRP, this neuropeptide is present in substantial amounts in skeletal tissue, including the periosteum and bone marrow [25], and it plays a role in osteoblastic activity, inhibiting osteoclastogenesis. In the particular case of CDF, patients have lowered CGRP levels [30]. As a result, CGRP fails to effectively interfere with osteoclastogenesis and bone tissue modulation, hence contributing to the physiopathology imbalances observed at the articular level [31].

### 1.6. Genetics

Nowadays, a substantial number of molecular and cellular pathways are considered to contribute to Charcot neuropathic arthropathy. The scientific community has recently started investigating the genetic mechanisms involved in this process [32]. Broadly, the motivation behind these undertakings is the hope to uncover the means by which we can explain several apparent clinical dissimilarities noticed between patients that otherwise present seemingly identical phenotypes [33]. That only a tiny percentage of patients diagnosed with diabetic neuropathy develop CNA suggests the possible involvement of genetic factors [34]. Several studies have examined the association between OPG gene polymorphisms and Charcot neuroarthropathy. Mrozikiewicz-Rakowska et al. conducted a study on 77 Charcot patients, 243 non-Charcot diabetic subjects with neuropathy, and 986 non-diabetic controls [35]. The authors pinpointed meaningful differences regarding allele distribution frequencies in the case of diabetic patients with symptomatic CNA, especially interesting with regard to the genes encoding RANK, RANKL, and OPG. In particular, the variations in allele frequencies in the OPG gene indicated further probable links between the RANK/RANKL/OPG signaling and TNFα inflammatory pathways [35].

### 1.7. Clinical Features and Classifications

**Figure 2:**
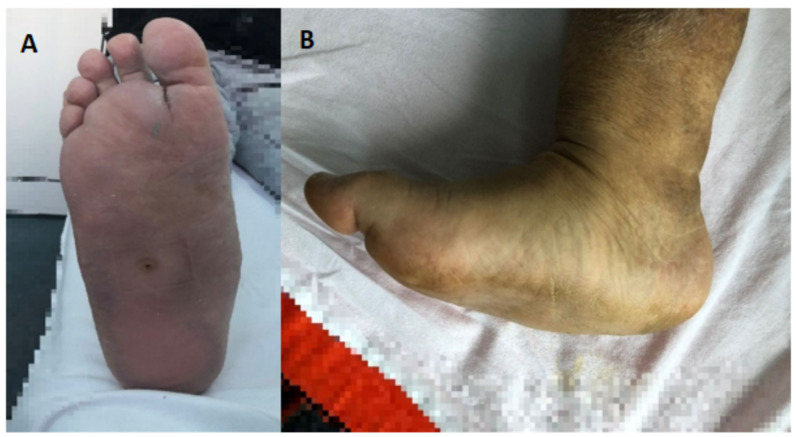
Typical aspect for Charcot foot deformities, Dr. Ion Cantacuzino Hospital Photo Archive; (**A**) dorsal view; (**B**) medial view.

Although numerous clinical signs and symptoms are described in the specialized literature, patients commonly seek medical assistance when presented with an abrupt onset of unilateral warmth, redness, and edema over the foot or ankle, which frequently includes a history of moderate trauma (Figure 2). The temperature of the afflicted foot may be several degrees higher than the contralateral foot and may also feel noticeably warmer to the touch [11]. Acute inflammatory attacks may occasionally occur, though individuals may present with a slowly developing arthropathy that evolves over months or years of insidious tumescence of the foot. The tarsal and tarsometatarsal joints, alongside the metatarsophalangeal joints and the ankle, are considered the most often affected joints in patients diagnosed with diabetes [11].

The clinical management of Charcot neuroarthropathy is elaborate. Unfortunately, the pathology is remarkably similar to an ankle sprain, cellulitis, venous thrombosis, inflammatory arthritis, and other frequently asymptomatic conditions, posing significant diagnostic challenges [36]. The collapsing of the midfoot arch and distinctive bony prominences unusually positioned are distinguishing signs of foot involvement, as the osseous prominences on the plantar aspect can result in pressure ulcerations, although in most cases, the skin is usually unaffected. However, foot ulceration and early neuroarthropathy can coexist, ulceration being a result of the foot deformity brought on by the late illness [3,11].

Regarding laboratory testing, a complete blood count (CBC), white blood cell count (WCC) differential count, and tests for renal function, such as blood urea nitrogen and creatinine, should all be performed in a patient suspected of having neuropathic arthritis. Additional testing is essential to rule out other differential diagnoses and can vary based on the initial clinical presentation. For example, in order to rule out vasculitic or rheumatic etiologies, further testing could include anti-nuclear antibodies, anti-nuclear extractable, anti-citrulline, c-ANCA, p-ANCA, cryoglobulin, or rheumatoid factor [3,18].

**Figure 3:**
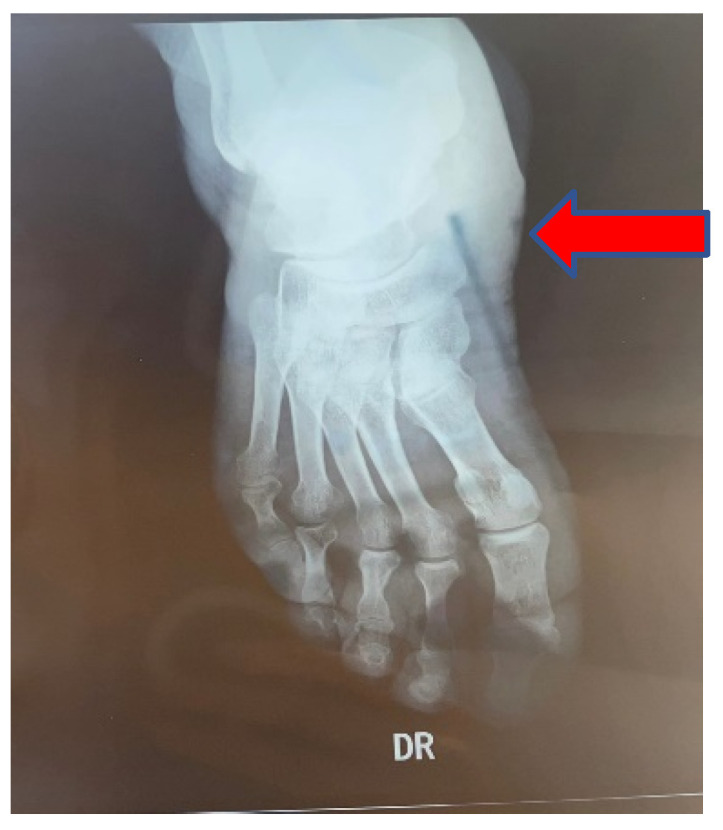
Radiograph of the right foot in a diabetic patient involving zone II according to the Sanders–Frykberg Classification, Dr. Ion Cantacuzino Hospital Photo Archive.

A comprehensive characterization of CNA can be achieved by employing two key classification algorithms. One of them, which uses an anatomically based approach that separates the foot into five primary zones depending on the joints involved, is the Sanders–Frykberg classification (Figure 3). The Eichenholtz classification, on the other hand, is a clinically based system that explains the progression of the illness and suggests a course of therapy based on related clinical and radiographic characteristics [18]. Based on the involved anatomical regions, the Sanders–Frykberg classification includes five models, regarded as patterns I through V. The first pattern describes the involvement of the phalanges, interphalangeal, and metatarsophalangeal joints.

The Eichenholtz classification based on clinical and radiological signs is most frequently utilized in the specialized literature as well as in clinical practice. Mild inflammation, soft tissue edema, and typical X-ray imaging results are all characteristic of stage 0; magnetic resonance imaging results may reveal abnormalities such as microfractures, bone marrow edema, or bone contusions. Early detection in this stage and prompt medical care might halt the disease’s progression and stop foot deformation [11,36].

Severe inflammation, soft-tissue edema, deviant X-rays with macro-fractures, accompanied by abnormal magnetic resonance imaging results revealing macro-fractures and bone marrow edema are the indicative findings for Eichenholtz Stage 1, articular dislocation being one of the promoters of osseous tissue resorption [36]. Stage 2 represents the coalescence phase, marking the end of bone resorption: clinical signs of inflammation are decreased, and remodeling processes are initiated, including fracture repair and debris resorption [18,36]. Stage 3 signifies comprehensive bone remodeling with osseous tissue rebuilding. Moreover, it corresponds to the installation of the chronic phase of CN, characterized by pressure ulcer formation secondary to a significant alteration of the foot’s arch. Bony deformities may be stable or unstable, and radiological imaging may demonstrate a mature fracture callus and diminished sclerosis [18,36].

**Aim of the study**: This systematic review aims to establish if there are, and which are the novel biomarkers that are predictive for diabetic Charcot foot.

## 2. Material and Methods

**Figure 4:**
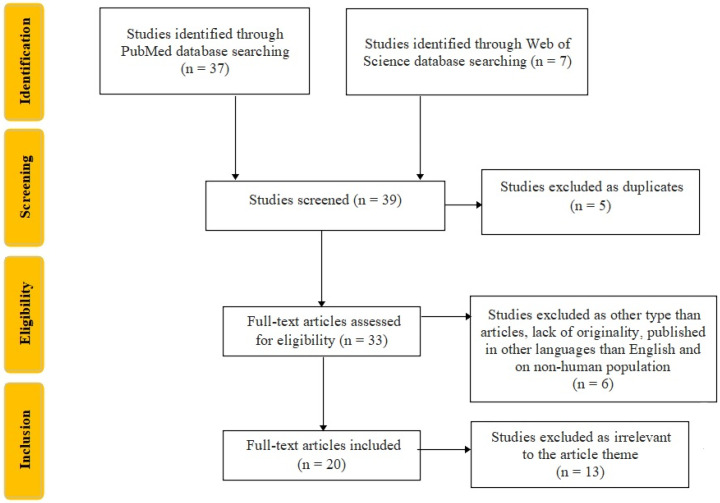
Flowchart of the study selection process according to PRISMA recommendations.

We developed an easily reproducible protocol for our study following the recommendations of Preferred Reporting Items for Systematic Reviews and Meta-Analyses (PRISMA) for the systematic review protocol checklist. Furthermore, we used the Population, Intervention, Comparison, Outcome, and Study Design (PICOS) strategy to guide our study rationale and to conduct a clear, useful, and systematic literature search. We searched using the criterion “biomarkers AND Charcot’s foot”, and included only full-text articles, both clinical trials and randomized controlled trials, published in English, and identified 33 articles on MEDLINE and seven articles on the Web of Science database. The inclusion criteria were original articles on the human population, published in English, while the exclusion criteria were duplicates, articles that lack originality, published in languages other than English, and on non-human populations. Two researchers, TS and AB, extracted the included studies’ titles and abstracts, screened for relevance for the present study theme, and selected the relevant ones by cross-screening, resulting in the inclusion of 20 articles, as seen in Figure 4.

## 3. Results

### 3.1. The Inflammatory Cascade

In Charcot diabetic foot, the chronic pro-inflammatory markers include interleukin-1β, interleukin 6, tumor necrosis factor-α, and increased receptor activation of nuclear factor-K β ligand, which is imbalanced with its receptor and osteoprotegerin [3,12] (Table 1).

There are reports of an increased level of IL-6 (Δvalue: 10.04 pg/mL, *p* = 0.049) and, respectively, AGEs (Δvalue: 2.5 ng/mL, *p* = 0.002) in patients with acute CDF symptoms (<3 months) in CDF versus the healthy foot, as venous-arterial flux, while there was no difference for free soluble RANKL (fsRANKL), OPG, IL-8, soluble receptor of AGE (sRAGE), or AGEs [15,37]. On the other hand, a decrease has been reported for fsRANKL at follow-up (*p* = 0.002), and an increment in Δ (fsRANKL/OPG ratio) at follow-up between DM+CDF versus DM-CDF (−2.9 versus −0.1, *p* = 0.046) [37] (Table 1).

When comparing patients with CDF versus patients with DM and without DM, the inflammatory markers tendency is to show increased levels of IL-6, IL-1β, TNF-α [16,38,39], OPG [16], and serum RANKL (sRANKL) [7], and decreased values for fsRANKL [16] (Table 1).

Also, when comparing CDF to patients with neuropathy and DM, RANKL and OPG have higher levels in the first two groups than in the last one [40]. At two years follow-ups, OPG is higher in patients with DM compared to CDF and healthy individuals and, respectively, higher than at the initial visit [20]. RANKL is higher in patients with CDF compared to patients with neuropathy or healthy controls [41]. It is important to emphasize that after three months of casting therapy, there was encountered a decrease in TNF-α and IL-6 [16] (Table 1).

Consistent off-loading leads to the suppression of Wnt-signaling by a receptor for advanced glycation end products proliferation, which maintains hyperglycemia and, consecutively, favors osteoclastogenesis with a reduction in RAGE, leading to increased AGEs levels that influence the RANKL from the system [3]. When comparing patients with CDF versus patients with DM and without DM, plasma RANKL levels are higher in the first group than in healthy subjects (*p* = 0.3) and, also, in the second group as compared to healthy control subjects (*p* < 0.001), with similar values between first two groups (*p* = 0.007) [38]. The OPG–RANKL ratio had similar values between patients with CDF, when compared to patients with DM and, respectively, without DM both at inclusion and two years of follow-up [20]. Bruhn-Olszewska et al. reported a decreased OPG–RANKL ratio between patients with CDF versus patients with DM [40] (Table 1).

The depletion of the vasoactive intestinal peptide with relatively normal substance P maintains the inflammation at the articular level [3] (Table 1).

Reducing calcitonin gene-related peptide and nitric oxide synthase leads to osteoclastogenesis and bone resorption [3] (Table 1).

Laboratory values, including the inflammatory markers, C-reactive protein, and erythrocyte sedimentation rate (ESR), are normal in the absence of infection signs [3]. Reported data shows an increased C-RP level in patients with CDF compared to patients without CDF both with DM and without DM (5.4 versus 3.7 mg/L versus 0.8 mg/L, *p* = 0.007) [16]. On the other hand, Hingsammer et al. also reported elevated C-RP, ESR, and white blood cell count even in the acute/subacute phase compared to the chronic phase of CDF [42]. There are also a few cases when a dissociation between clinical (increased skin temperature) and the lack of systemic markers (a normal to a slight increase in C-RP levels, normal WCC, and a mild increase in ESR) is present, such as the one reported by Petrova et al. [43]; or when there is no difference for C-RP and ESR when comparing CDF treated with pamidronate versus placebo at baseline and at the 3-, 6-, 9-, and 12-month follow-ups, such as the one reported by Jude et al. [44] (Table 1).

Uccioli et al. reported no difference in CD40, CD80, and CD86 expression in CDF, and the increment from acute CDF goes back to normal after the acute phase, but they observed an increased resistance of monocytes, in the acute CDF, to serum withdrawal-induced apoptosis [38] (Table 1).

Mabilleau et al. reported increased CD14-positive cells for patients with CDF compared to patients with DM and healthy controls [39] (Table 1).

Rizzo et al. reported a higher reactivity against native collagen type I (CI) and type II (CII) in DCF as well as autoantibodies against post-translationally modified (ox-PTM) CI and CII, as compared to patients with T2DM or healthy controls, except for antibodies against glycated-CI and peroxynitrite modified CI [45]. No difference was observed when comparing patients with CDF to patients with DM for native, glycated, hypochlorous acid, hydroxyl radical, and peroxynitrite-modified CII, respectively. In contrast, significantly increased binding was observed comparing CDF patients with DM patients, except peroxynitrite-modified CII [45] (Table 1).

### 3.2. Pro-Inflammatory Changes in the Immune Phenotype and the Whole Methylome of Monocytes

Monocytes, as shown by fluorescence-activated cell sorter (FACS) study in acute CDF, produce TNF-α, IL-1β, and IL-6 as compared to patients with DM or normal controls; also, when activated by lipopolysaccharide (LPS) from Escherichia coli 0111/B4, they produce produced more TNF-α, IL-1β, and IL-6, but less IL-4 and IL-10, than patients with DM or healthy controls; but in both cases they decrease after the recovery from acute phases [38].

Gene-mapped differential methylation is higher in CDF patients, as compared with patients with DM, including hypermethylation (86%), suggesting that circulating monocytes seem to be ‘ready’ for differentiation; second, that they may be involved in monocyte differentiation into osteoclasts, while regarding DNA methylation and gene expression in CDF patients only the PPP2R5D gene had a cis association with expression [46].

### 3.3. Calcium and Bone Turnover Parameters

Jansen et al. reported a decrease in CTX (carboxy-terminal collagen crosslinks), at baseline versus follow-up (387 ± 136 versus 95 ± 83 ng/L, *p* < 0.001) in the DM+CDF group and (305 ± 141 versus 90 ± 40 ng/L, *p* < 0.001) in the DM-CDF group, but with no difference for osteocalcin (16.9 ± 5.7 versus 14.7 ± 11.5 μg/L, *p* = 0.153) in the DM+CDF group or, respectively, (11.1 ± 4 versus 10.4 ± 3.6 μg/L, *p* = 0.695) in the DM-CDF group [15].

Petrova et al., comparing patients with CDF to patients with DM and without DM, reported an increased value for C-terminal telopeptide (0.24 versus 0.12 versus 0.15 μg/L, *p* = 0.004) alongside bone alkaline phosphatase (b-ALP) (16.4 versus 13.6 versus 10.1 μg/L, *p* = 0.006) and no statistically significant decrease in tartrate-resistant acid phosphatase (3.9 versus 2.7 UI/L, *p* = 0.126) [16] (Table 2).

Jirkovská et al. reported, when comparing CDF to healthy controls, increased b-ALP, I crosslinked C-telopeptides (ICTP), serum hydroxyproline, and urine hydroxyproline/creatinine, and what is more, a significant correlation between the T-score of stiffness and ICTP (r = ±0.729, *p* < 0.001) and between the T-score of stiffness and S-hydroxyproline (r = ±0.55, *p* < 0.05) [16] (Table 2). Jade et al. reported b-ALP and dehydroxypyridinoline crosslinks significantly reduced at 4- and 24-week follow-ups, but the difference was not maintained at the 3-, 9-, and 12-month follow-ups when comparing patients with CDF treated with pamidronate with patients with CDF treated with placebo [44].

### 3.4. Genotype Predisposition

Some genotypes lead to dysregulation of the RANK–OPG axis through single nucleotide polymorphisms (SNPs), such as G1181C and T245G SNPs, or thymine–thymine polymorphism of the 1217 SNP and the 245 SNP [3].

Brun-Olszewska et al. reported, when comparing CDF patients with neuropathy patients and, respectively, patients with DM, no genotype changes for three out of ten analyzed loci: OPG 950T/C, RANK 421C/T, and RANK 575C/T, while genotype changes were present for C genotype in the case of OPG 1181C/G, for CC genotype in OPG 6890A/C, for the identical genotype in OPG 245T/G and OPG 1217 C/T in patients with CDF [20]. Moreover, for RANK, the TT genotype for 290C/T and 643C/T, and the CC genotype for 693G/C were identified [40].

The linkage disequilibrium (LD) in CDF revealed higher values between OPG 245T/G and OPG 1217C/T (r2 = 0.99) and a weaker association between OPG 1181G/C and 950T/C (r2 = 0.51); while for RANKL, a higher value between RANKL 693G/C and 290C/T (r2 = 0.89) and a weaker association between RANKL 693G/C and 643C/T (r2 = 0.52), while the LD analysis of OPG and RANKL polymorphisms did not show disequilibrium [40].

Hierarchical clustering of SNPs in the OPG, RANKL, and RANK genes identified OPG 245T/G and OPG 1217C/T with a nearly identical pattern of distribution; meanwhile, RANK 421C/T and OPG 6890A/C also cluster together with OPG 245T/G and 1217C/T, and the CDF has the most considerable number of patients in the third cluster, and an equal number of patients with DM in the second cluster [40].

SaiPrathiba et al. reported that when comparing patients with CDF and with diabetic neuropathy to normal patients, for RANKL 643 C/T and 693 C/G polymorphism the “CC” genotype was more frequent among normal subjects; for RANKL 643 C/T, the homozygous genotype “TT” of the minor allele as well as the heterozygous genotype “CT”, greater frequency of the “T” and “G” alleles; while for RANKL 693 C/G, the homozygous genotype “GG” as well as the heterozygous genotype “GC” were less frequent [41]. Moreover, there were no significant differences for RANKL (643 C/T and 693 C/G) polymorphism between patients with diabetic neuropathy and CDF [41].

### 3.5. Paraclinical Investigations

Jansen et al. reported that comparison at a 10-year follow-up with DXA scanning between patients with DM and CDF and, respectively, without CDF, showed no differences in total hip [37,45] and calcaneal bone mineral density (BMD), and an increase in BMD of lumbar L2–L4 of +0.036 g/cm^2^ (+2.9%) in the DM+CDF group, versus +0.125 g/cm^2^ (+10.1%) in the DM-CDF group [37] (Table 3).

Christensen et al. reported a difference in calcaneal BMD in patients with DM and chronic CDF when compared to the healthy contralateral calcaneal BMD (*p* < 0.01) [48] (Table 3).

When comparing CDF and non-CDF patients, Jirkovská et al. reported a difference in the T-score of stiffness of the calcaneus, alongside a lower mean T-score of stiffness of the calcaneus; and a lower mean T-score of BMD in the femoral neck, but with no difference in the mean T-score of BMD in the lumbar spine [47]. Moreover, for CDF, the mean T-score of stiffness of the calcaneus was lower than both the mean BMD T-scores in the lumbar spine (*p* < 0.001) and in the femoral neck (*p* < 0.05); meanwhile, a comparison of BMD in the lumbar spine and in the femoral neck revealed lower values in the latter as compared to controls [47] (Table 3).

Comment et al. reported a successfully established decision tree employing 3-foot BMD-based parameters as a possible beneficial prognostic imaging marker for CDF [49].

Folestad et al. reported sclerostin, Dkk-1, Wnt-1, and Wif-1 levels at initial visits higher in patients with DM versus CDF and healthy subjects but similar in CDF and healthy subjects; and, respectively, at two-year follow-up as higher for sclerostin and Wnt-1; and with no difference for Dkk-1, but with higher values for Wif-1 in patients with DM versus CDF [20] (Table 3).

In vivo corneal confocal microscopy (CCM) of the sub-basal nerve plexus (SNP) identified a decreased corneal nerve fiber length, a decreased corneal fiber nerve and, respectively, branch density and decreased corneal nerve connecting points (connections/mm) for CDF as compared to controls [50,51]. Moreover, by immunostaining, intraepidermal nerve fiber, Meissner corpuscle as well as Meissner cell density are decreased, alongside the nodal length-to-diameter ratio, the paranodal length, and the fraction of long nodes of Ranvier. However, the density of bundles with myelinated nerve fibers is increased in patients with Charcot type 1 foot compared to control patients [52].

## 4. Summary

The Charcot diabetic foot is still one of the terrifying complications for diabetes patients, resulting in amputations and even death. Correct and early diagnosis of this illness is still an issue that requires additional research. Considering that the quality of life of patients with Charcot’s foot is highly influenced by the presence of this debilitating osteoarthropathy, knowledge and prevention of the disease progression are essential factors that must be pointed out with great attention from the first signs of the disease.

The current review shows that previous studies have highlighted two significant classes of biomarkers involved in the pathogenesis of Charcot’s foot—on the one hand, pro-inflammatory markers and, on the other hand, markers of osteolysis/osteosynthesis. A comprehensive synthesis of the known and of novel biomarkers can be seen in Table 4.

Our study identified that the essential inflammatory biomarkers were TNF-α, IL-6, and IL-1β; the osseous markers were RANK/RANKL and osteoprotegerin, Sclerostin, Dkk-1, and Wnt-1.

Taking into consideration that in daily rheumatological practice there are enough biosimilar/biological therapies used against these molecules, which have been demonstrated as efficient in the treatment of inflammatory rheumatic pathology, the extension of the indication and usage of these medications in the early stages of the CDF must be encouraged, thus providing an early means of counteracting the inflammation process and the inflammatory signaling that leads to joint destruction.

Likewise, the osteolysis and osteosynthesis mechanisms activated by inflammation can be limited by using the latest generation of bisphosphonate type (antiresorptive therapy) and newer biological therapy molecules.

Recognizing the first signs of Charcot arthropathy using biomarkers and even the co-administration of anti-cytokine therapy with anti-osteoporotic therapy in the early stages is more than justified.

## Figures and Tables

**Table 1:** Summary of the included studies’ reported bio-markers that take part in the Charcot diabetic foot’s inflammatory process (↑ represents increment of the biomarker levels, ↔ represents represents mainitainance of the biomarker levels).

	IL-1 β	IL-6	TNF-α	RANKL	OPG	AGE	fsRANKL	sRAGE	**fsRANKL/OPG Ratio**	**C-RP**	**ESR**	**WCC**
**Schmidt et al. [3]**	↑	↑	↑	↑	↑			↑				
**Molines et al. [12]**	↑		↑	↑	↑							
**Jansen et al. [15]**		Δ value: 10.04 pg/mL, *p* = 0.049		No difference	No difference	Δ value: 2.5 ng/mL, *p* = 0.002						
**Petrova et al. [16]**	0.27 vs. 0.2 vs. 0.18, *p* = 0.254	3.3 vs. 2 vs. 1.4, *p* = 0.002	1.3 vs. 1 vs. 0.8, *p* = 0.01	0.29 vs. 0.41 vs. 0.13, *p* = 0.915			5.4 vs. 4.4 vs. 2.9, *p* < 0.001			5.4 vs. 3.7 vs. 0.8, *p* = 0.007		
**Jansen et al. [37]**					5.93 vs. 7.71, *p* = 0.812		0.04 vs. 0.68, *p* = 0.002	1593 vs. 399, *p* = 0.005	(−2.9 vs. −0.1, *p* = 0.046			
**Uccioli et al. [38]**	0.6 ± 0.3 vs. <0.125, *p* < 0.005	15.3 ± 7.4 vs. 6.7 ± 3.5, *p* < 0.05	5.2 ± 3.2 vs. 2.6 ± 1.2, *p* < 0.05	↑, *p* < 0.001								
**Mabilleau et al. [39]**			4.3 ± 0.9 vs. 1.93 ± 0.8, *p* = 0.009									
**Bruhn-Olszewska et al. [40]**				1.01 ± 1.45 vs. 2.66 ± 1.74 vs. 0.5 ± 0.43 pmol/L, *p* < 0.001	7.36 ± 4.1 vs. 6.29 ± 1.68 vs. 4.77 ± 2.38 pmol/L, *p* < 0.001				↑			
**Folestad et al. [20]**				↑, *p* = 0.004	↑, *p* < 0.001				↔			
**SaiPrathiba et al. [41]**				8.9 vs. 7.4 vs. 5.12 ng/mL, *p* = 0.008								
**Hingsammer et al. [42]**										34.7 vs. 9.5 mg/L, *p* = 0.01	25.9 vs. 18.3 mm/h, *p* = 0.02	11.8 vs. 8.2 10^9^/L, *p* = 0.01
**Petrova et al. [43]**										5.8 (5–11) vs. ≤5 mg/L	↑	normal
**Jude et al. [44]**										No difference	No difference	

**Table 2:** Summary of the included studies’ reported calcium and bone turnover parameters that can be used as bio-markers in the Charcot diabetic foot (↓ represents decrement of the biomarker levels).

	X	Osteocalcin	C-Terminal Telopeptide	b-ALP	Tartrate-Resistant Acid Phosphatase
**Petrova et al. [16]**			0.24 vs. 0.12 vs. 0.15 μg/L, *p* = 0.004	16.4 vs. 13.6 vs. 10.1 μg/L, *p* = 0.006	3.9 vs. 3.9 vs. 2.7 UI/L, *p* = 0.126
**Jansen et al. [37]**	387 ± 136 vs. 95 ± 83 ng/L, *p* < 0.001	16.9 ± 5.7 vs. 14.7 ± 11.5 μg/L, *p* = 0.153			
**Jirkovská et al. [47]**				↓, *p* < 0.03	

**Table 3:** Summary of the included studies’ reported paraclinical investigation parameters that can be used as bio-markers in the Charcot diabetic foot (↑ represents increment of the biomarker levels, ↔ represents represents mainitainance of the biomarker levels, ↓ represents decrement of the biomarker levels).

	Total Hip BMD	L2-L4 BMD	Calcaneal BMD	Femoral Neck BMD	Sclerostin	Dkk-1	Wnt-1	Wif-1
**Folestad et al. [20]**					↑	↑	↑	↑
**Jansen et al. [37]**	↔, *p* = 0.294	+0.036 g/cm^2^ (+2.9%) vs. +0.125 g/cm^2^ (+10.1%)						
**Jirkovská et al. [47]**		−0.57 ± 1.28 vs. −0.91 ± 0.84, *p* > 0.05	−3.00 ± 1.39 vs ±2.36 ± 1.12; *p* < 0.01	−1.58 ± 1.24 vs. −0.76 ± 0.98, *p* < 0.05				
**Christensen et al. [48]**			↓, *p* < 0.01					

**Table 4:** The summarization of the biomarkers known for Charcot’s foot. C-RP = C-reactive protein; ESR = erythrocyte sedimentation rate; WCC = white blood cell count; IL-1 β = interleukin 1 beta; IL-6 = interleukin 6; TNF-α = tumor necrosis factor alpha; RANKL = receptor activator of nuclear factor kappa-Β ligand; OPG = osteoprotegerin; AGE = advanced glycation end products; fsRANKL = free soluble receptor activator of nuclear factor-κB; sRAGE = soluble receptors of advanced glycation end products; b-ALP = bone alkaline phosphatase; Dkk-1 = Dickkopf-related protein 1; Wnt-1 = wingless/integrated family member 1; Wif-1 = Wnt inhibitory factor 1; BMD = bone mineral density.

Common Biomarkers	Novel Biomarkers
C-RP [15,16,43,44]	RANKL [20,35,40,41]
ESR [3,42,43,44]	OPG [20,35]
WCC [3,18]	AGE [3,15,37]
IL-1 β [12,16,38]	fsRANKL [15,16,37]
IL-6 [12,15,16,37,38]	sRAGE [15,37]
TNF-α [16,24,38,39]	Osteocalcin [15,37]
	C-terminal telopeptide [16]
	b-ALP [16,44,47]
	Tartrate-resistant acid phosphatase [16]
	Sclerostin [20,21,22,23]
	Dkk-1 [20,21,22,23]
	Wnt-1 [20,21,22,23]
	Wif-1 [20,21,22,23]
	Total hip BMD [37,45]
	L2-L4 BMD [37,47]
	Calcaneal BMD [37,38,47,48]
	Femoral neck BMD [47,48,49]

## Data Availability

Not applicable.
